# Supplementary material for: Trends of non-vaccination, under-vaccination and missed opportunities for vaccination (2003–2014) amongst children 0–23 months in Kenya
Source: PLOS Glob Public Health. 2024 Feb 6;4(2):e0002906. doi: 10.1371/journal.pgph.0002906 (PMC10846728; doi:10.1371/journal.pgph.0002906)
Supplement: Attachment — Submitted filename: Response to Reviewers.docx [file pgph.0002906.s001.docx]

Christopher Ochieng’ Odero,

P. O. Box 51048 – 00200,

Nairobi.

August 22, 2023

Dr. Steve Zimmerman

Staff Editor, PLOS Global Public Health

**RE: Response to reviewers - Manuscript ID: PGPH-D-23-01014**

**Manuscript title: Trends of Non-vaccination, Under-vaccination, and Missed Opportunities for Vaccination (2003-2014) Amongst Children 0-23 Months in Kenya.**

We extend our sincere appreciation for your meticulous review of our manuscript and the invaluable insights you and the reviewers have provided. We have carefully considered both your comments and recommendations, and we respectfully offer our responses below.

**Reviewer’s comment**: The reviewer requests additional information about how the weights were created to deal with the uneven distribution.

**Author’s response**: We have applied sampling weights to address the inherent disparities in the distribution of our data. These weights were meticulously formulated to rectify variations in the likelihood of selecting sampling units, households, and individuals, while accounting for the response rate per stratum, which encompassed counties and enumeration areas. To elaborate, the household weight for a specific household is computed as the reciprocal of its household selection probability, multiplied by the reciprocal of the household response rate within the stratum. Subsequently, the individual weight is derived by multiplying the household weight by the reciprocal of the individual response rate within the same stratum. The concept of response rate groups, where households and individuals are categorized into sample strata to calculate response rates, is pivotal in KDHS surveys. This approach ensures that our analysis encompasses a representative sample of the population, and it upholds the statistical integrity of our findings.

**Reviewer’s comment**: The reviewer wonders if the 3 tables could be condensed.

**Author’s response**: We have judiciously structured the presentation of tables in alignment with the KDHS tables and variables, allowing for seamless comparisons and facile references for readers. This formatting decision was made with the intent to maximize reader convenience and provide an accessible interface for cross-referencing, especially considering the amalgamation of data from the three surveys. Therefore, we deem it prudent to maintain the tables in their current format to facilitate efficient understanding and interpretation.

**Reviewer’s commen**t: The reviewer would prefer the introduction to be more concise.

**Author’s response**: We concur with the perspective shared by the Journal editors that our introduction is concise and appropriately informative. We believe that the introduction concisely encapsulates the essential context required to introduce our study, thereby ensuring clarity without redundancy.

In conjunction with this letter addressing each raised point from both your esteemed self and the esteemed reviewer(s), we have attached a marked-up version of our manuscript, highlighting the revisions made, alongside an unmarked version of the revised manuscript for your perusal.

We are eagerly anticipating your endorsement of our manuscript for publication. Your guidance and insights have been invaluable in enhancing the quality and rigor of our work.

Best regards,

Christopher Ochieng’ Odero,
