# Supplementary material for: Trends of non-vaccination, under-vaccination and missed opportunities for vaccination (2003–2014) amongst children 0–23 months in Kenya
Source: PLOS Glob Public Health. 2024 Feb 6;4(2):e0002906. doi: 10.1371/journal.pgph.0002906 (PMC10846728; doi:10.1371/journal.pgph.0002906)
Supplement: Attachment — Submitted filename: Response to Reviewers - 18 Dec 2023.docx [file pgph.0002906.s002.docx]

Christopher Ochieng’ Odero,

P. O. Box 51048 – 00200,

Nairobi.

December 21, 2023

Dr. Jianhong Zhou

Staff Editor

PLOS Global Public Health

**RE: Response to reviewers - Manuscript ID: PGPH-D-23-01014**

**Manuscript title: Trends of Non-vaccination, Under-vaccination, and Missed Opportunities for Vaccination (2003-2014) Amongst Children 0-23 Months in Kenya.**

We extend our sincere appreciation for your meticulous review of our manuscript and the invaluable insights you and the reviewers have provided. We have carefully considered both your comments and recommendations, and we respectfully offer our responses below. Also see the manuscript with tracked changes.

1. **Reviewer comment**: Line 38, “51.3” should be “51.3%”?

**Response**: This has been corrected to read 51.3%. See line 38.

2. **Reviewer** **comment**: The introduction section of the article should show the main research question and purpose. Currently it is unclear and too long.

**Response**: The whole introduction section has been revised. It is now shorter and more precise. The main research question and purpose have been well elaborated. Please see pages 3-8.

3. **Reviewer comment**: Line 73-75, “In 1993….” should be formatted the English correctly. And the language of the entire article needs to be revised by native speaker further.

**Response**: This concern has been addressed as part of the revisions to the whole introduction section.

4. **Reviewer comment**: Line 159-168, Please show more details on the sample size calculation methods. What is the calculated formula and parameters you used?

**Response**: We have provided the formula used in the sample size calculation as well as the assumptions that were made. Please refer to page 10 and 11 for more details.

5. **Reviewer comment**: What is the statistical method you used to arrive at the results for tables 1-3? Is it a regression? Because there are 3 columns of results for 2003-2014 but only one p value.

**Response**: The focus of this analysis is on multi-year trends of non-vaccination, under-vaccination and missed opportunity for vaccination. The statistical analysis conducted is The Cochrane-Armitage trend test. Each table (1-3) represent each of non-vaccination, under-vaccination and missed opportunities for vaccination between 2003, 2008/9, and 2014. For each table, The Cochrane-Armitage trend test was calculated and corresponding p value presented.

6. **Reviewer comment**: Table 1-3, All tables need to be presented in a three-line table that conforms to the format of the publication. And please double-check the numbers in all the tables, for example, Table 1 has a problem with the number 3.5 (6.5-16.8) for mothers aged 45-49, in 2014 column.

**Response**: We have revised the presentation of tables 1-3 as recommended. In addition, we have gone through the numbers in each table (1-3) and verified that they are correct. The 95% CI of mothers aged 45-49 in the 2014 column has been corrected. See page 14.

7. **Reviewer comment**: How can the results section adequately support the findings and conclusions of the study when there is no figure with the data presented? Please add some figures.

**Response**: Our results are presented in alignment with the DHS results for ease of reference and comparison for readers. This formatting decision was made with the intent to maximize reader convenience and provide an accessible interface for cross-referencing, especially considering the amalgamation of data from the three surveys. However, we have added figure 1 (page 21) in response to the reviewer’s comment.

In conjunction with this letter addressing each raised point from both your esteemed self and the esteemed reviewer(s), we have attached a marked-up version of our manuscript, highlighting the revisions made, alongside an unmarked version of the revised manuscript for your perusal.

We are eagerly anticipating your endorsement of our manuscript for publication. Your guidance and insights have been invaluable in enhancing the quality and rigor of our work.

Best regards,

Christopher Ochieng’ Odero,
